# Post-discharge clinical, laboratory and radiographic features of coronavirus disease 2019 (COVID-19) patients at university hospitals in Japan

**DOI:** 10.20407/fmj.2021-024

**Published:** 2022-05-25

**Authors:** Toshiharu Sasaki, Yoko Toyama, Tomoya Horiguchi, Masaya Hibino, Sei-ichiro Tsuzuki, Masamichi Hayashi, Yohei Doi, Mitsunaga Iwata, Kazuyoshi Imaizumi, Masato Inaba

**Affiliations:** 1 Department of Infectious Diseases, Fujita Health University, School of Medicine, Toyoake, Aichi, Japan; 2 Department of Respiratory Medicine, Fujita Health University, School of Medicine, Toyoake, Aichi, Japan; 3 Department of Emergency and General Internal Medicine, Fujita Health University, School of Medicine, Toyoake, Aichi, Japan; 4 Division of Infectious Diseases, University of Pittsburgh, School of Medicine, Pittsburgh, Pennsylvania, USA

**Keywords:** Long COVID, Computed tomography, Alanine aminotransferase, SARS-CoV-2, Cohort study

## Abstract

**Objectives:**

Coronavirus disease 2019 (COVID-19) has affected nearly half million people in Japan. However, information on the prolonged symptoms as well as laboratory and radiographic findings after hospital discharge remains limited.

**Methods:**

We retrospectively collected the symptoms, laboratory test results, and chest imaging results of COVID-19 patients at the time of the hospital admission and the ambulatory visits after discharge at two university hospitals between July and December 2020.

**Patients:**

A total of 126 COVID-19 patients, including of 88 with mild to moderate disease and 38 with severe to critical disease, were included. The time between symptom onset and the first outpatient visit was 46 days (Interquartile range, 39 to 55).

**Results:**

At the ambulatory visits, 36.5% of patients had at least one symptom. The most frequent symptom was shortness of breath (12.8%), followed by cough (11.1%), and fatigue (8.8%). Of 120 patients with post-discharge laboratory test results, 27 patients (22.5%) had abnormal alanine aminotransferase levels, and 35 patients (29.1%) had lymphocytopenia, including 24 and 27 mild and moderate patients. Of 122 patients with post-discharge chest computed tomography (CT) scans, 105 (83.3%) had abnormal findings. This abnormality was found in both mild to moderate and severe patients.

**Conclusions:**

Shortness of breath, abnormal liver function test results and chest CT images often persisted for at least one month after discharge, even when symptoms were mild or moderate during hospitalization.

## Introduction

The ongoing coronavirus disease 2019 (COVID-19) pandemic has impacted over two hundred million people worldwide. Its clinical presentations range from asymptomatic to severe and critical disease requiring intensive care. Common laboratory features include lymphopenia, elevated liver function enzymes and coagulation abnormalities, whereas computed tomography of the lungs may reveal pneumonia even in asymptomatic patients.^1^

As COVID-19 patients recover from acute illness, prolonged and chronic symptoms are observed among some of them.^2^ In a report from Italy, only 13% of hospitalized patients experienced complete resolution of COVID-19-related symptoms after discharge, whereas a study from China showed that at least one symptom persisted in 76% of hospitalized patients after discharge.^3,4^ In Japan, follow-up phone interviews revealed persistence of various symptoms, ranging from cough, shortness of breath to fatigue, four months after discharge from hospital.^5^ Prolonged symptoms have also been observed among those with mild symptoms who did not require hospitalization.^6^

Japan was one of the countries hit early in the pandemic with nearly two million confirmed COVID-19 cases, but information on post-discharge course of patients that include laboratory and radiographic findings remains limited. Here, we describe the incidence and nature of prolonged symptoms and their association with laboratory and radiographic abnormalities of COVID-19 patients who were hospitalized, discharged, and returned to ambulatory clinics at a university health system in Japan.

## Materials and Methods

### Patient enrollment

Patients aged 18 or greater who were admitted with a confirmed diagnosis of COVID-19 to one of the two hospitals of a university health system in central Japan and presented to ambulatory clinics for a follow-up visit between July 1 and December 31, 2020 were included in this retrospective analysis. The initial diagnosis of COVID-19 was based on the public guideline in the country.^7^ During the study period, COVID-19 patients were strongly encouraged to be admitted to hospitals or observation facilities for isolation regardless of the severity of symptoms. The workup at this hospital system for COVID-19 patients generally consisted of laboratory tests (complete blood count and expanded chemistry panel) and computed tomography (CT) of the chest at the time of hospital admission, and the same workup at the time of the ambulatory visit scheduled approximately one month after discharge for those who had respiratory symptoms or pneumonia on CT images during hospitalization. All ambulatory patients also underwent temperature measurement and screening of COVID-19-related symptoms including cough, shortness of breath, dysosmia and dysgeusia.

### Date collection

Severity of illness, administration of antiviral agents or steroids, laboratory values (complete blood count with differentials, liver function tests, creatinine and C-reactive protein), and chest CT images were reviewed. Mild disease was defined by SpO_2_ ≥94% at ambient air in the absence of pneumonia based on imaging, and moderate disease was defined by SpO_2_ ≥94% at ambient air in the presence of pneumonia on imaging. Severe and critical diseases were defined by SpO_2_ <94% or requirement for supplemental oxygen, and need for intensive care, respectively. The highest level of disease severity during the course of hospitalization was recorded for this purpose.^8^ Liver function abnormality was defined as alanine aminotransferase (ALT) values >40 IU/L, and lymphocytopenia was defined by absolute lymphocyte counts ≤1,500 cells/mL.^9^ In the outpatient setting, corresponding data obtained at the first ambulatory visit after hospital discharge were collected from the electronic medical records.

### Ethical approval

This study was approved by the Ethics Committee of the Faculty of Medicine, Fujita health University, Aichi, Japan (approved number HM20-337).

## Results

### Patient characteristics in this study

A total of 126 patients who were hospitalized for COVID-19 and followed up in the ambulatory clinic after discharge were included. Among them, 88 had mild or moderate disease and 38 had severe or critical disease during their hospitalization. The patient characteristics are shown in Table 1. The median length from symptom onset to admission was 6 days (IQR, 4.0 to 8.0) overall, 5 days (IQR, 4,0 to 8.0) for mild/moderate cases and 6 days (IQR, 4.3 to 8.8) for severe/critical cases, respectively. The median length from symptom onset to the first ambulatory visit was 46 days (IQR, 39.0 to 55.0) overall, 44 days (IQR, 37.0 to 53.0) for mild/moderate cases and 50 days (41.0 to 58.0) for severe/critical cases, respectively. Fatty liver was the most common comorbidity in mild/moderate cases, while hypertension was the most common comorbidity, followed by fatty liver and dyslipidemia in severe/critical cases. Ten patients were current smokers, and 40 patients had history of smoking, including 29 mild/moderate cases and 11 severe/critical cases. Fever was present in 120 patients at admission, and 20 of 38 severe/critical patients needed supplemental oxygen at admission. The length of hospital stay was 10 days (IQR, 8.0 to 13.8) overall, 9 days (IQR, 7.0 to 11.3) in mild/moderate cases, and 13.5 days (IQR, 11.0 to 16.0) in severe/critical cases. Two patients required mechanical ventilation. The most common treatment directed at COVID-19 in mild/moderate cases was favipiravir, whereas steroids (dexamethasone or methylprednisolone) were frequently used in severe/critical cases.

### Comparison of clinical symptoms at admission and the first ambulatory visit after discharge

The most common symptoms at the time of hospitalization were fever, followed by fatigue, and shortness of breath. At the time of the first ambulatory visit, 46 patients (29 with mild/moderate disease and 17 with severe/critical disease) still had at least one symptom. As shown in Table 2, shortness of breath, cough, and fatigue persisted in 16 patients (12.8%) (10 [11.5%] in mild/moderate cases and 6 [15.8%] in severe/critical cases), 14 patients (11.1%) (9 [10.2%] in mild/moderate cases and 5 [13.2%] in severe/critical cases), and 11 patients (8.8%) (7 [8.0%] in mild/moderate cases and 4 [10.5%] in severe/critical cases), respectively. Between discharge and the post-discharge ambulatory visit, hair loss, graying of hair and herpes zoster were newly observed in 5, 1, and 1 patients, respectively.

### Comparison of laboratory test results at admission and the first ambulatory visit after discharge

Laboratory test results at the time of admission and the first ambulatory visit after discharge are shown in Table 3. Post-discharge laboratory test results were available for 120 patients. Of those, 35 (29.1%) and 27 (22.5%) patients had abnormal lymphocyte and ALT levels at the outpatient visits, respectively. Twenty-four of 27 patients who had elevated ALT levels were under the age of 65 and had mild to moderate disease during hospitalization. None of these patients had subjective symptoms suggestive of liver damage, and 13 of them had no symptoms at the outpatient visit. All patients with abnormal AST values at the outpatient visit underwent abdominal ultrasound examination or abdominal CT scan, which revealed bright liver consistent with fatty liver in 19 of them (70.4%). Upon interviewing, none of these patients admitted to increased drinking or taking new medications. The elevation of ALT levels persisted during the follow-up period (median, 55 days after symptom onset; IQR, 37.5 to 81). Among patients with lymphocytopenia at the outpatient visit, one patient experienced infection (varicella zoster) after hospital discharge.

### Comparison of CT imaging results at admission and the first ambulatory visit after discharge

Of the 126 patients who underwent Chest CT scan at the time of admission, 117 (93.6%) patients (79 [90.8%] in mild/moderate cases and 38 [100%] in severe/critical cases) had images consistent with pneumonia. The most common finding on CT scan was bilateral ground-glass opacity (GGO) (64%), followed by bilateral GGO and consolidation (17.6%) (Table 4). Chest CT scans were performed in 122 patients at the outpatient visits, and 105 patients had images consistent with pneumonia. Among them, pneumonia had improved since the time of hospital admission in 95 patients (Table 5). Of these 105 patients, 84 (80%) had no respiratory symptoms.

## Discussion

While persistent COVID-19 symptoms and their sequelae are increasingly reported, long-term impact of COVID-19 on laboratory and radiographic findings in the convalescence period remains unclear. To address this knowledge gap, we compared the symptoms, laboratory values and imaging results of COVID-19 patients between the time of hospitalization and at post-discharge ambulatory visits.

Overall, one-third of patients had persistent COVID-19 symptoms at the outpatient visits. The rate was lower than those reported in other studies,^3–5^ likely because most of the patients in our cohort had mild to moderate disease. Furthermore, COVID-19 patients were encouraged to be admitted to hospitals or observation facilities for isolation regardless of the severity of symptoms, making it possible that the severity of disease among patients in our cohort were somewhat lower even in the same severity category compared with other reports. The most common symptoms that persisted was shortness of breath, followed by cough and fatigue. The persisted symptoms were found not only among sever/critical patients but also among mild/moderate patients. This might suggest the needs for careful follow-up for patients with even mild/moderate symptoms during the illness. Most patients had pneumonia on chest CT at the time of hospitalization, yet the majority of them had no residual respiratory symptoms at the outpatient visits. American College of Radiology recommends against universal chest CT imaging and suggests that indication should be guided clinically.^10^ Our findings support these recommendations.

As for laboratory values, AST and ALT values were elevated in about 30% of patients upon hospitalization, and about one in five patients had persistently abnormal levels at the outpatient visit. None of the patients with transaminase elevation had signs or symptoms consistent with hepatitis. Elevation of liver transaminase levels during the acute phase of COVID-19 has been well documented. The potential mechanisms for this phenomenon include infection of the hepatocytes expressing angiotensin-converting enzyme 2 (ACE2), “bystander hepatitis” as has been observed with other viral infections, and medications, but the damage is generally considered to be self-limiting.^11,12^ In our cohort, some patients had ALT elevation over more than two months from the onset of COVID-19. None of the patients with ALT elevation at the outpatient visit had received any additional medications after discharge and the levels of CRP were low. In addition, bright liver was common in these patients, and increased expression of ACE2 in non-alcoholic fatty liver disease has been reported previously.^13^ These observations suggest that the main mechanism of elevation of liver enzyme levels is not “bystander hepatitis” or medication, but rather the effect of COVID-19 itself. Additionally, patients with abnormal liver enzyme levels in our cohort had no subjective symptoms suggestive of liver damage and about half of them did not have any symptoms at the outpatient visits despite the fact that nearly 90% of patients experienced at least mild symptoms during admission. In addition, the elevated values of AST and ALT was found in even mild/moderate patients. Taking these aspects into consideration, continued surveillance of liver function, regardless of the severity of the diseases, may be prudent for such patients as the long-term impact of COVID-19 to their underlying liver disease is yet to be determined.^12^ Lymphocytopenia also persisted after discharge in a fifth of the patients, but it is reassuring that only one patient had an episode of infection, which was self-limited in nature.

A strength of our study is that symptomatic COVID-19 patients were routinely hospitalized per government policy, making it easier to ascertain their severity of disease and conduct initial workup including chest CT imaging. We also brought them back in person for follow-up visits instead of remote or virtual visit, which allowed us to conduct bloodwork and chest CT scans in most of them. The limitations include the preponderance of those with mild and moderate disease and small number of patients with severe or critical disease. However, mild disease accounts for >80% of COVID-19 globally,^14^ therefore the study may represent an unbiased picture of the long-term impact of this disease. Also, the follow-up period was limited in this study, and we intend to continue following up on patients with persistent symptoms or abnormal laboratory findings. In addition, since our small sample size was small, we performed only descriptive analysis. Instead, we reviewed the chart of patients intensively.

In conclusion, a minority of COVID-19 patients may present with long-term laboratory abnormalities, in particular elevated liver transaminase levels in addition to the well-recognized persistent clinical symptoms even in mild and moderate cases. How these patients can be identified early and whether they lead to clinically relevant adverse outcomes in the longer term requires further investigation.

## Figures and Tables

**Table1:** Patient characteristics

Number of patients	Overall	Mild/moderate	Severe/critical
	126	88	38
Baseline and demographic			
Age, years (IQR)	59 (48.3, 73.0)	56 (44.8, 67.8)	64 (56.3, 76.8)
Female, n (%)	38 (30.2)	30 (34.1)	8 (21.1)
Body mass index (IQR)	24.5 (21.7, 27.5)	24.9 (21.7, 28.0)	23.9 (22.2, 26.1)
Days between symptom onset and admission, median days (IQR)	6.0 (4.0, 8.0)	5.0 (4.0, 8.0)	6.0 (4.3, 8.8)
Days between symptom onset and first ambulatory visit, median days (IQR)	46.0 (39.0, 55.0)	44.0 (37.0, 53.0)	50.0 (41.0, 58.0)
Comorbidities			
Hypertension, n (%)	37 (29.4)	20 (22.7)	17 (44.7)
Dyslipidemia, n (%)	24 (19.0)	14 (15.9)	10 (26.3)
Fatty liver, n (%)	41 (32.5)	31 (35.2)	10 (26.3)
Diabetes mellitus, n (%)	20 (15.9)	14 (15.9)	6 (15.8)
Asthma, n (%)	7 (5.6)	5 (5.7)	2 (5.3)
Malignancy, n (%)	5 (4.0)	2 (2.3)	3 (7.9)
Lung disease other than chronic obstructive pulmonary disease, n (%)	3 (2.4)	1 (1.1)	2 (5.3)
Chronic obstructive pulmonary disease, n (%)	3 (2.4)	1 (1.1)	2 (5.3)
Immunosupressant use, n (%)	2 (1.6)	2 (2.3)	0 (0)
Chronic kidney disease, n (%)	2 (1.6)	0 (0)	2 (5.3)
Ischemic heart disease, n (%)	7 (5.6)	4 (4.5)	3 (7.9)
Chronic heart failure, n (%)	2 (1.6)	2 (2.3)	0 (0)
Steroid use	1 (0.8)	1 (1.1)	23 (60.5)
Smoking			
Current smoking, n (%)	10 (8.5)	9 (11)	1 (2.8)
Past smoking, n (%)	40 (33.9)	29 (35.4)	11 (30.6)
Vital signs at the admission			
Body temperature, °C (IQR)	37.5 (36.7, 38.2)	37.3 (36.7, 38.2)	37.7 (37.0, 38.3)
Fever, n (%)	120 (97.6)	84 (95.5)	36 (94.7)
SpO_2_, % [IQR]	96.0 (94.3, 98.0)	97.0 (95.0, 98.0)	94.5 (92.0, 97.0)
Oxygen requirement on the admission, n (%)	20 (15.8)	0 (0)	20 (52.6)
Clinical course			
Length of hospital admission, days (IQR)	10.0 (8.0, 13.8)	9.0 (7.0, 11.3)	13.5 (11.0, 16.0)
ICU admission, n (%)	6 (4.8)	0 (0)	6 (13.2)
Mechanical ventilation, n (%)	2 (1.6)	0 (0)	2 (5.2)
Treatment for COVID-19			
Remdesivir, n (%)	13 (10.0)	2 (2.3)	11 (28.9)
Steroid, n (%)	28 (22.2)	5 (5.7)	23 (60.5)
Ciclesonide, n (%)	2 (1.6)	0 (0)	2 (5.3)
Favipiravir, n (%)	20 (15.9)	14 (15.9)	6 (15.8)
Heparin, n (%)	3 (2.4)	1 (1.1)	2 (5.3)

**Table2:** Clinical symptoms at admission and the first ambulatory visit

	Overall (N=126)		Mild/moderate (N=88)		Severe/critical (N=38)	
	At admission	First ambulatory visit	At admission	First ambulatory visit	At admission	First ambulatory visit
Any of the symptoms below	125 (99.2)	46 (36.5)	87 (98.8)	29 (32.9)	38 (100)	17 (44.7)
Shortness of the breath, n (%)	52 (41.9)	16 (12.8)	31 (36.0)	10 (11.5)	21 (55.3)	6 (15.8)
Chest pain, n (%)	7 (5.6)	0 (0)	6 (6.8)	0 (0)	1 (2.6)	0 (0)
Diarrhea, n (%)	25 (19.8)	0 (0)	21 (23.9)	0 (0)	4 (10.5)	0 (0)
Cough, n (%)	30 (23.8)	14 (11.1)	23 (26.1)	9 (10.2)	7 (18.4)	5 (13.2)
Fatigue, n (%)	80 (64.5)	11 (8.8)	54 (62.8)	7 (8.0)	26 (68.4)	4 (10.5)
Dysgeusia/dysosmia, n (%)						
Dysgeusia	21 (16.7)	6 (4.8)	16 (18.2)	3 (3.4)	5 (13.2)	3 (7.9)
Dysosmia	5 (4.0)	3 (2.4)	5 (5.7)	3 (3.4)	0 (0)	0 (0)
Both	13 (10.3)	2 (1.6)	10 (11.4)	2 (2.3)	3 (7.9)	0 (0)
Nausea, n (%)	8 (6.3)	0 (0)	8 (9.1)	0 (0)	0 (0)	0 (0)
Vomiting, n (%)	5 (4.0)	0 (0)	5 (5.7)	0 (0)	0 (0)	0 (0)
Hair loss, n (%)	0 (0)	5 (4.0)	0 (0)	3 (3.4)	0 (0)	2 (5.3)
Gray hair, n (%)	0 (0)	1 (0.8)	0 (0)	1 (1.1)	0 (0)	0 (0)
Varicella zoster, n (%)	0 (0)	1 (0.8)	0 (0)	1 (1.1)	0 (0)	0 (0)

**Table3:** Laboratory tests at admission and the first ambulatory visit after discharge

	Overall		Mild/moderate		Severe/critical	
	At admission	First ambulatory visit	At admission	First ambulatory visit	At admission	First ambulatory visit
Number of patients	126	120	88	84	38	36
Complete blood count						
White blood cell count, ×10^3^/mL (IQR)	5.0 (4.0, 6.2)	5.9 (5.1, 6.9)	4.7 (3.8, 6.2)	5.8 ^(^5.0, 6.8)	5.3 (4.5, 6.6)	6.1 (5.4, 7.2)
Lymphocyte count, ×10^3^/mL (IQR)	0.95 (0.75, 1.3)	1.8 (1.4, 2.2)	1.1 (0.86, 1.4)	1.8 (1.4, 2.2)	0.76 (0.6, 0.96)	1.9 (1.6, 2.2)
Lymphocyte count ≤1500/mL, n (%)	109 (86.5)	35 (29.1)	74 (84.1)	27 (32.1)	35 (92.1)	8 (22.2)
Hemoglobin, g/dL (IQR)	14.6 (13.4, 15.9)	14.2 (13.1, 15.3)	14.7 (13.5, 15.9)	14.3 (13.1, 15.2)	14.4 (13.4, 15.9)	14.2 (13.1, 15.4)
Liver function						
AST, IU/dL (IQR)	36.0 (24.0, 51.8)	22.0 (19.0, 29.3)	32.0 (22.0, 46.3)	22.0 (19.0, 31.5)	44.5 (31.0, 68.5)	22.5 (19.0, 26.0)
ALT, IU/dL (IQR)	30.0 (18.0, 49.0)	21.0 (14.0, 37.3)	29.5 (16.8, 50.8)	24.0 (14.0, 42.3)	31.0 (22.0, 42.8)	20.0 (15.8, 23.5)
ALT >40 IU/dL, n (%)	42 (33.3)	27 (22.5)	56 (63.6)	24 (20.0)	28 (73.7)	3 (8.3)
Total bilirubin, mg/dl (IQR)	0.6 (0.5, 0.8)	0.6 (0.5, 0.8)	0.6 (0.4, 0.7)	0.6 (0.5, 0.8)	0.6 (0.5, 0.7)	0.7 (0.5, 0.8)
γ-GTP, IU/L (IQR)	43.5 (24.0, 90.1)	76.0 (64.0, 91.3)	42.5 (23.8, 77.3)	77.0 (65.5, 90.5)	46.5 (26.5, 146.5)	69.0 (60.5, 88.5)
ALP, IU/L (IQR)	72.5 (56.3, 89.5)	29.0 (22.0, 56.0)	72.0(59.5, 87.0)	29.0 (20.5, 55.0)	75.5 (52.5, 106.7)	29.5 (22.0, 54.5)
Renal function						
BUN, mg/dL (IQR)	12.9 (10.1, 16.1)	13.5 (11.0, 16.0)	12.8 (10.3, 15.7)	13.7 (11.0, 16.1)	13.2 (10.1, 16.1)	13.0 (10.1, 15.4)
Creatinine, mg/dL (IQR)	0.83 (0.68, 0.97)	0.77 (0.66, 0.89)	0.82 (0.67, 0.96)	0.73 (0.66, 0.87)	0.86 (0.70, 1.0)	0.80 (0.67, 0.90)
Creatinine kinase, U/L (IQR)	75.0 (54.0, 120.8)	76.5 (55.3,99.0)	71.5 (51.0, 104.0)	76.0 (55.0,99.0)	96.0 (64.3, 194.3)	78.0 (56.0, 92.0)
CRP, mg/dL (IQR)	4.2 (1.0, 7.7)	0.10 (0.03, 0.31)	2.8 (0.6, 6.7)	0.10 (0.03, 0.27)	6.3 (3.3, 10.3)	0.08 (0.04, 0.48)
LDH, IU/L (IQR)	249.0 (206.3, 346.0)	187.0 (161.0, 210.3)	239.0 (190.0,300.0)	178.5 (158.8, 203.0)	338.5 (246.5, 412.3)	202.0 (175.5, 243.8)

**Table4:** Chest CT images at admission

	Overall	Mild/moderate	Severe/critical
Pneumonia on chest CT, n (%)	117 (93.6)	79 (90.8)	38 (100)
Bilateral GGO and consolidation	22 (17.6)	12 (13.8)	10 (26.3)
Bilateral GGO	80 (64.0)	52 (59.8)	28 (73.7)
Unilateral GGO	10 (8.0)	10 (11.4)	0 (0)
Unilateral consolidation	3 (2.4)	3 (3.4)	0 (0)
Unilateral GGO and consolidation	2 (1.6)	2 (2.2)	0 (0)

GGO, ground glass opacity.

**Table5:** Chest CT images at first ambulatory visit

	Overall	Mild/moderate	Severe/critical
CT at outpatient visits, n (%)	122 (96.8)	84 (95.4)	3 (100.0)
Days between symptoms onset and chest CT, days (IQR)	51.0 (40.0, 62.5)	51.0 (40.0, 72.0)	51.5 (40.8, 59.5)
Pneumonia on chest CT, n (%)	105 (83.3)	69 (78.4)	36 (94.7)
Improvement of chest CT, n (%)	95 (75.4)	60 (67.8)	35 (92.2)
